# Hybrid sequencing recovers highly contiguous genome assemblies from eight cultured *Planktothrix rubescens* isolates from Lake Zurich

**DOI:** 10.1128/mra.00820-26

**Published:** 2026-10-26

**Authors:** Cyrill Hofer, Deborah Knapp, Thomas Posch, Adrian-Stefan Andrei, Jakob Pernthaler

**Affiliations:** 1 Limnological Station, Department of Plant and Microbial Biology, University of Zurich 27217, Kilchberg, Switzerland; 2 Eawag, Swiss Federal Institute of Aquatic Science and Technology 28499, Dübendorf, Switzerland; California State University San Marcos, San Marcos, California, USA

**Keywords:** *Planktothrix rubescens*, cyanobacteria, complete genome, hybrid assembly, nanopore sequencing, Illumina sequencing, Lake Zurich, cultured isolates, genomics

## Abstract

We report highly contiguous genome assemblies from eight cultured *Planktothrix rubescens* strains isolated from Lake Zurich, Switzerland. Hybrid Illumina and Nanopore sequencing produced high-quality, near-complete, near-contaminant-free assemblies, including seven single-contig genomes, four flagged as circular.

## ANNOUNCEMENT

*Planktothrix rubescens* is a filamentous freshwater cyanobacterium that can produce microcystins, potent hepatotoxins that threaten lakes as drinking-water sources and recreational sites (1). In oligo-mesotrophic lakes such as Lake Zurich, low-light-adapted *P. rubescens* can form dense populations in the metalimnion, making complete reference genomes valuable for studying toxin biosynthesis, strain diversity, and ecological adaptation (2).

The sequenced strains included the axenic reference strain A7 (3), isolated from Lake Zurich in 1993 by A. E. Walsby, and seven non-axenic isolates. The latter were obtained from Lake Zurich by plankton net sampling at 0–15 m depth in May–June 2018 (47°18′16″N, 8°34′31″E), single-filament picking with a glass micropipette, washing through three successive drops of sterile-filtered (0.2 µm) lake water, and reinoculation into Cyanomedium (4). Cultures were maintained under laboratory conditions (~6 µmol photons m^−2^ s^−1^, 18°C) and transferred to fresh medium approximately every 6 weeks prior to DNA extraction. For each culture, 25 mL was filtered onto 5 µm polycarbonate membranes (Nuclepore Track-Etch Membrane, 47 mm; Whatman). Cut filters containing filaments were transferred to 1× PBS and heated at 80°C for 6 min. DNA extraction followed a modified protocol incorporating the METIS step, developed for high-molecular-weight DNA extraction from cyanobacteria (5). Briefly, heat-treated samples were processed with proteinase K, sodium dodecyl sulfate (SDS), and lysozyme. Lysates were clarified by sequential centrifugation, and DNA was precipitated in isopropanol. During precipitation, pigmented mucilage-containing fractions were repeatedly removed and replaced with fresh isopropanol until the solution cleared and DNA became visible. DNA was pelleted, resuspended in nuclease-free water, and purified using AMPure XP beads (Beckman Coulter). Libraries were prepared and multiplexed using the Illumina TruSeq DNA Nano kit without modification and the Oxford Nanopore Ligation Sequencing gDNA Native Barcoding Kit (SQK-NBD114.24), without DNA shearing or size selection for the ONT libraries. Libraries were sequenced on an Illumina NovaSeq 6000 platform (2 × 150 bp; FGCZ, Zurich) and an Oxford Nanopore MinION Mk1D using an R10 FLO-MIN114 flow cell (Limnological Station, Zurich). Nanopore reads were basecalled, demultiplexed, and adapter-trimmed using Dorado v7.11.2 (Oxford Nanopore Technologies) with the super-accurate model v5.5.0. Illumina reads were quality-trimmed with BBDuk from BBMap v39.26 (6) (qtrim=rl trimq=18) and screened for vector and adapters (*k*=21), while Nanopore reads were filtered with Chopper v0.7.0 (7) (-q 10 -l 500) and adapter-trimmed with Porechop_ABI v0.5.1 (8). Sequencing generated medians of 16.3 Gb of Illumina short-read data and 0.67 Gb of Nanopore long-read data (median N50: 7.18 kb) per strain. Reads were mapped against 75 publicly available *Planktothrix* genomes using BBMap v39 (6) (kfilter = 31, subfilter = 15, maxindel = 80) for Illumina reads and minimap2 v2.28 (9) (-x map-ont) for Nanopore reads. Unmapped reads were discarded, retaining median per-strain data sets of 12.9 Gb of Illumina data (Q39) and 0.23 Gb of Nanopore data (Q19; N50, 11.7 kb) for hybrid assembly. Hybrid assemblies were generated with Unicycler v0.4.8 (10) and manually curated by iterative removal of short contigs guided by CheckM2 v1.0.2 (11) estimates, preserving completeness while reducing contamination. Illumina read counts were obtained with FastQC v0.11.9 (12), while Nanopore read counts and read N50 values were calculated with NanoStat v1.6.0 (13). GC content, CDS, rRNA, and tRNA counts were extracted from Prokka v1.14.6 (14) annotations. Default parameters were used unless otherwise specified.

Seven assemblies were single contig, including four closed circular genomes; one remained in four contigs (Table 1). This genome collection provides a valuable resource for studying toxin biosynthesis, strain diversity, and ecological adaptation in *P. rubescens*.

**TABLE 1:** *Planktothrix rubescens* strains from Lake Zurich^*a*^

Strain	Accession	Circ.	Ctgs. (*n*)	Comp. (%)	Cont. (%)	Size (Mb)	CDS (*n*)	5S (*n*)	16S (*n*)	23S (*n*)	tRNA (*n*)	GC (%)	Reads (*n*)	ONT N50 (bp)
ZS_A7	GCA_984783665	Yes	1	99.98	0.00	5.40	4819	3	4	4	42	39.48	120,873,822 (Ill.) 11,270 (ONT)	20,465
ZS_3A4	GCA_984783925	Yes	1	99.99	0.03	5.43	4835	3	4	4	42	39.51	25,939,422 (Ill.) 50,642 (ONT)	9,156
ZS_3C4	GCA_984783935	Yes	1	99.97	0.00	5.43	4845	3	4	4	42	39.50	80,018,090 (Ill.) 133,352 (ONT)	5,595
ZS_4B4	GCA_984783585	Yes	1	99.95	0.00	5.42	4807	3	4	4	42	39.48	69,766,194 (Ill.) 42,581 (ONT)	6,496
ZS_3C1	GCA_984783895	No	1	99.99	0.01	5.40	4821	3	4	4	42	39.51	97,384,004 (Ill.) 7,557 (ONT)	14,857
ZS_5C5	GCA_984783575	No	1	99.95	0.00	5.38	4799	3	4	4	42	39.50	106,501,368 (Ill.) 15,444 (ONT)	14,180
ZS_5C6	GCA_984783635	No	1	99.95	0.00	5.49	4872	3	4	4	42	39.52	70,047,462 (Ill.) 37’301 (ONT)	7,156
ZS_4D3	GCA_984783605	No	4	99.98	0.01	5.42	4831	3	4	4	42	39.51	92,604,192 (Ill.) 16,634 (ONT)	18,218

^*a*^ Circ., circular; Ctgs., contigs; Comp., completeness; Cont., contamination; Ill., Illumnia.

## Data Availability

The sequencing reads and genome assemblies are available through the European Nucleotide Archive (ENA) under BioProject PRJEB114346/study accession ERP194653 (*Planktothrix rubescens* of Lake Zurich). Individual genome accession numbers are provided in Table 1. The corresponding Prokka annotation outputs and associated genome files for all eight strains are available through the project GitHub repository (https://github.com/cyhofe/Planktothrix-rubescens-genomes-Lake-Zurich) and are permanently archived on Zenodo at https://doi.org/10.5281/zenodo.22208404.
